# Circ_0114428 knockdown inhibits ROCK2 expression to assuage lipopolysaccharide-induced human pulmonary alveolar epithelial cell injury through miR-574-5p

**DOI:** 10.1186/s12576-023-00891-3

**Published:** 2024-01-31

**Authors:** Jing Zhao, Qin Zhao, Qiuxia Duan

**Affiliations:** 1 Department of Critical Care Medicine, Yantai City Yantaishan Hospital, Keji Avenue, Laishan District, Yantai, 10087 Shandong China; 2 Emergency Internal Medicine Department, Weifang People’s Hospital, Weifang, Shandong China; 3 Department of Critical Care Medicine, The Third People’s Hospital of Qingdao, No. 29 Yongping Road, Licang District, Qingdao, 266000 Shandong China

**Keywords:** ALI, circ_0114428, miR-574-5p, ROCK2, LPS

## Abstract

**Background:**

Sepsis-induced acute lung injury (ALI) accounts for about 40% of ALI, accompanied by alveolar epithelial injury. The study aimed to reveal the role of circular RNA_0114428 (circ_0114428) in sepsis-induced ALI.

**Methods:**

Human pulmonary alveolar epithelial cells (HPAEpiCs) were treated with lipopolysaccharide (LPS) to mimic a sepsis-induced ALI cell model. RNA expression of circ_0114428, miR-574-5p and Rho-associated coiled-coil containing protein kinase 2 (ROCK2) was detected by qRT-PCR. Protein expression was checked by Western blotting. Cell viability, proliferation and apoptosis were investigated by cell counting kit-8, 5-Ethynyl-29-deoxyuridine (EdU) and flow cytometry analysis, respectively. The levels of pro-inflammatory factors were detected by enzyme-linked immunosorbent assay (ELISA). Oxidative stress was analyzed by lipid peroxidation Malondialdehyde (MDA) and Superoxide Dismutase (SOD) activity detection assays. The interplay among circ_0114428, miR-574-5p and ROCK2 was identified by dual-luciferase reporter, RNA pull-down and RNA immunoprecipitation assays.

**Results:**

Circ_0114428 and ROCK2 expression were significantly increased, but miR-574-5p was decreased in blood samples from sepsis patients and LPS-stimulated HPAEpiCs. LPS treatment led to decreased cell viability and proliferation and increased cell apoptosis, inflammation and oxidative stress; however, these effects were relieved after circ_0114428 knockdown. Besides, circ_0114428 acted as a miR-574-5p sponge and regulated LPS-treated HPAEpiC disorders through miR-574-5p. Meanwhile, ROCK2 was identified as a miR-574-5p target, and its silencing protected against LPS-induced cell injury. Importantly, circ_0114428 knockdown inhibited ROCK2 production by interacting with miR-574-5p.

**Conclusion:**

Circ_0114428 knockdown protected against LPS-induced HPAEpiC injury through miR-574-5p/ROCK2 axis, providing a novel therapeutic target in sepsis-induced ALI.

**Supplementary Information:**

The online version contains supplementary material available at 10.1186/s12576-023-00891-3.

## Highlights

1. Circ_0114428 was upregulated in the blood samples of sepsis patients.
2. LPS treatment increased circ_0114428 expression in HPAEpiCs.
3. Knockdown of circ_0114428 ameliorated LPS-induced lung cell injury.
4. Circ_0114428 modulated ROCK2 expression through miR-574-5p.

**Supplementary Information:**

The online version contains supplementary material available at 10.1186/s12576-023-00891-3.

## Introduction

Developing as a result of immune response to pathogen-induced infection, sepsis is a complicated disorder with a high mortality rate of ~ 25% worldwide [1, 2]. Sepsis is usually accompanied by the dysfunction of the cardiovascular system, liver, kidney as well as lung [3]. Sepsis-induced acute lung injury (ALI) is considered the leading cause of sepsis-caused death [4]. In terms of mechanism, lung macrophages and infiltrating neutrophils that mediate the inflammatory process during sepsis-induced ALI lead to oxidative stress and the activation of redox-sensitive transcription factors, further resulting in increased production of pro-inflammatory cytokines [5]. As reported, increased lung cell apoptosis is another cause of acute lung injury [6]. Finally, the activation of inflammatory and apoptotic signaling disrupts alveolar epithelial cells and increases epithelial permeability [7]. Therefore, understanding the inner mechanism of inflammation, apoptosis and oxidative stress in lung epithelial cells may be necessary to develop a therapeutic target for sepsis-induced acute lung injury.

As a brand-new regulator, circular RNA (circRNA) is an endogenous RNA that is generated by back-splicing with a covalent bond linking 5ʹ cap and 3ʹ polyadenylation tail. The rapid advancement in RNA-sequencing analyses catalyzes the investigation of circRNA functions in multiple diseases, such as cancers, cardiovascular diseases, neurological diseases and diabetes mellitus [8]. At the molecular level, circRNA functions by sponging microRNAs (miRNAs), sequestering proteins or interfering with pre-mRNA processing [9]. There are increasing studies about the role of circRNA in lung diseases since 2017 [10]. In particular, Li et al. identified 581 differently expressed circRNAs in ALI mice in comparison with healthy controlled mice and demonstrated that these circRNAs were mainly associated with some signaling pathways, such as focal adhesion, neurotrophin, and Wnt [11], which suggested the contribution of circRNAs to ALI development. Circ_0114428 is located in chr:91403041-91447927-, and consists of exons 2–4 of zinc finger protein 644 (ZNF644), and the published data have confirmed its upregulation in the serum samples of septic acute kidney injury [12]. However, whether circ_0114428 regulates sepsis-induced ALI has not been reported.

MiRNAs are a kind of small RNAs of 19–25 nucleotides that mainly function at the posttranscriptional level, serving important parts in cell proliferation, apoptosis, metabolism as well as immunity [13]. MiR-574-5p, a member of the miRNA family, is dysregulated in a variety of diseases [14–16]. A recent study has indicated that miR-574-5p is able to inhibit inflammatory response induced by lipopolysaccharide (LPS) through interaction with toll like receptor-4 (TLR4)/nuclear factor kappa B (NF-κB) pathway [17]. Besides, Wang et al. have deciphered that miR-574-5p is upregulated in sepsis survivors in comparison with nonsurvivors [18]. However, very few researches were performed on the inner mechanism of miR-574-5p regulating sepsis-induced ALI.

Thus, the study was designed to analyze the role of circ_0114428 in sepsis-induced ALI by using an ALI cell model, which was established by stimulating human pulmonary alveolar epithelial cells (HPAEpiCs) using LPS. As predicted by the circinteractome database (https://circinteractome.nia.nih.gov/index.html), circ_0114428 potentially combined with miR-574-5p. We subsequently determined whether miR-574-5p was involved in circ_0114428-mediated regulation in LPS-induced lung injury, and revealed the mechanism responsible for the contribution of circ_0114428/miR-574-5p axis to sepsis-induced ALI development.

## Materials and methods

### Patient samples

The study subjects included 23 sepsis induced-ALI patients (age: 40 to 65 years old; gender: 12 male and 11 female) and 19 healthy controls (age: 40 to 65 years old; gender: 10 male and 9 female). ALI patients did not receive any treatment before admission. These patients with sepsis were hospitalized owing to bacterial infections. Blood was collected from ALI patients on admission. After blood coagulation, serum is separated by centrifugation at 2000 r/min for 10 min and stored at − 80 °C for subsequent analysis of gene expression. All participants were recruited from Yantai City Yantai Mountain Hospital, and submitted the written informed consent. The patients with other severe clinical disorders or pregnant women were excluded. The Ethics Committee of Yantai City Yantai Mountain Hospital approved the study.

### Cell culture and treatment

HPAEpiCs were purchased from ScienCell Research Laboratories (San Diego, California, USA) and cultured in Dulbecco’s modified Eagle’s medium (DMEM; Lonza, Basel, Switzerland), which was added with 10% heat-inactivated fetal bovine serum (FBS; Lonza) and 1% penicillin/streptomycin (Procell, Wuhan, China), at 37 °C with 5% CO_2_. HPAEpiCs were passaged every 3 days during culture. HPAEpiCs were cultured for 12–16 h and then exposed to 1 mg/L LPS (Solarbio, Beijing, China) to establish sepsis-induced ALI cell model inferring to the previous reference [19].

### Cell transfection

In accordance with the guidebook of FuGENE6 Transfection Reagent (Roche, Basel, Switzerland), the cells were cultured for 12–16 h and then transfected with plasmids, miRNA mimics, miRNA inhibitors and small interfering RNAs (siRNAs). Gentle shaking was conducted during cell transfection. Cells were cultured for the defined time for the following studies. The overexpression plasmid of Rho associated coiled-coil containing protein kinase 2 (ROCK2) expression was built by introducing full-length coding sequence of ROCK2 into pcDNA 3.1 vector (pcDNA), termed ROCK2. Ribobio Co., Ltd. (Guangzhou, China) provided miR-574-5p mimics (miR-574-5p, 5ʹ-UGAGUGUGUGUGUGUGAGUGUGU-3ʹ), miR-574-5p inhibitors (anti-miR-574-5p, 5ʹ-ACACACUCACACACACACACUCA-3ʹ), siRNAs specific to circ_0114428 (si-circ_0114428, 5ʹ-GACTATGCACTCAGGTTTGAT-3ʹ) and ROCK2 (si-ROCK2, 5ʹ-GCGGATTCACTTGTAGGAACATATA-3ʹ), and matched controls (miR-NC, anti-miR-NC, si-NC and si-con). The full-length sequence of circ_0114428 and pCD5-ciR vector were used to achieve circ_0114428-overexpressed plasmid (circ_0114428) in Geneseed Co., Ltd. (Guangzhou, China).

### Quantitative real-time polymerase chain reaction (qRT-PCR)

RNA from the blood samples of sepsis patients and HPAEpiCs was extracted using RNA isolation reagent (Tsingke, Shanghai, China). 1 μg of RNA was subjected to incubation with 4 U RNase R (CWBIO, Beijing, China) to digest linear RNA. Synthesis of cDNA was conducted following the instruction of High-Capacity Reverse Transcription Kit (TaKaRa, Dalian, China). Then, Fast qPCR Mix (Tsingke) was used to analyze gene expression with a qRT-PCR machine (Stratagene, Santa Clara, CA, USA). Finally, circRNA/miRNA/mRNA expression was analyzed by the 2^−∆∆Ct^ method, with normalization to U6 (for miRNA) and glyceraldehyde 3-phosphate dehydrogenase (GAPDH) (for circRNA/mRNA). The relative primer sequences were displayed in Table 1.

**Table 1:** Primers sequences used for qRT-PCR

Name		Sequences (5ʹ-3ʹ)
circ_0114428	Forward	ACTATGCACTCAGGTTTGATT
	Reverse	CACCAGTAATATCGGTGTTTA
ZNF644	Forward	TCGCCATCTTAATGTCCC
	Reverse	TTCCTCCATCAACTTCTGTC
miR-574-5p	Forward	CGCGTGAGTGTGTGTGTGTGA
	Reverse	AGTGCAGGGTCCGAGGTATT
ROCK2	Forward	GAGCCTGAGTGCGGGTC
	Reverse	AAGCCATCCAGCAAGCTCTC
GAPDH	Forward	CAAATTCCATGGCACCGTCA
	Reverse	GACTCCACGACGTACTCAGC
U6	Forward	CTTCGGCAGCACATATACT
	Reverse	AAAATATGGAACGCTTCACG

### Subcellular fractionation assay

HPAEpiCs were detached using trypsin (Thermo Fisher, Waltham, MA, USA) after discarding the culture medium. The cells were rinsed using phosphate buffer solution prior to pelleting at a low speed. The follow-up procedures were performed following the instruction of PARIS™ Kit (Thermo Fisher). At last, circ_0114428 in cytoplasmic fraction or nuclear pellet was analyzed by qRT-PCR with the expression of U6 and GAPDH as references.

### Cell counting kit-8 (CCK-8) assay

HPAEpiCs in logarithmic growth were digested and passaged in 96-well plates, followed by treatment with LPS, plasmids, miRNA mimics, miRNA inhibitors, siRNAs and matched controls according to the aforementioned methods. Afterward, the cells were exposed to DMEM containing CCK-8 reagent (Beyotime, Shanghai, China). Four-hour culture later, samples were analyzed by an enzyme-linked immune detector (Thermo Fisher) with a wavelength of 450 nm.

### 5-Ethynyl-29-deoxyuridine (EdU) assay

HPAEpiCs with various treatments as mentioned above were allowed to grow in 6-well plates for 48 h. Then, the cells were detached and passaged into 96 well plates, which were added with EdU labeling medium. DNA synthesis was analyzed with an EdU staining kit (Ribobio) as per the guidebook. Finally, EdU-positive cells from a high-power field (100x) microscope (Olympus, Tokyo, Japan) were captured.

### Flow cytometry analysis

After LPS exposure and cell transfection, HPAEpiCs were harvested for apoptotic rate analysis using a commercial apoptosis detection kit (Elabscience, Shanghai, China). In brief, the cells were resuspended in Binding Buffer, and then reacted with Annexin V-FITC and propidium iodide. The results were analyzed using a flow cytometer (Thermo Fisher) with CytExpert software.

### Western blotting analysis

Protein samples were prepared using RIPA buffer (Thermo Fisher). After measuring protein concentration, the protein lysates were loaded onto polyacrylamide gels, and wet-transferred on to nitrocellulose membranes. Then, the primary antibody for BCL2-associated x protein (Bax; Cat #33-6400; 1:1000; Thermo Fisher), cleaved-caspase-3 (Cat #PA5-114687; 1:2000; Thermo Fisher), ROCK2 (Cat #PA5-78290; 1:3000; Thermo Fisher) or GAPDH (Cat #39-8600; 1:1000; Thermo Fisher) was used to incubate the membranes. After proper washing, the membranes were probed with secondary antibodies. Enhanced chemiluminescence (KeyGen, Nanjing, China) was used to develop protein blots. Protein expression was normalized to GAPDH.

### Inflammatory cytokines analysis

The supernatant of HPAEpiCs was collected after LPS exposure and/or cell transfection. Then, the concentrations of interleukin-6 (IL-6) as well as tumor necrosis factor-α (TNF-α) in the cell supernatant were analyzed using enzyme-linked immunosorbent assay (ELISA) kits (Beyotime), and the assay procedures were performed inferring to the guidebooks.

### Analysis of oxidative stress

The assays regarding the detection of Malondialdehyde (MDA) and Superoxide Dismutase (SOD) activity were performed to analyze oxidative stress. For lipid peroxidation MDA assay, about 2 × 10^6^ HPAEpiCs were harvested after LPS treatment and/or cell transfection. The cells were washed using PBS and lysed using MDA lysis buffer (Abcam, Cambridge, MA, USA). Cell supernatant was collected by centrifugation, and incubated with thiobarbituric acid (Abcam) at 95 °C for 1 h, followed by analysis using a microplate reader (Thermo Fisher). For SOD activity assay, HPAEpiCs were collected and lysed using lysis buffer (Abcam), followed by centrifugation to harvest cell supernatant. The following assay was performed inferring to the instruction of a SOD activity assay kit (Abcam). The output of samples was determined on a microplate reader (Thermo Fisher).

### Dual-luciferase reporter assay

Online databases circinteractome (https://circinteractome.nia.nih.gov/index.html) and starbase (http://starbase.sysu.edu.cn/agoClipRNA.php?source=mRNA) were used to predict the complementary sites of miR-574-5p with circ_0114428 and ROCK2. The sequences of circ_0114428 and the 3ʹ-untranslated region (3ʹUTR) of ROCK2 contained the complementary sites were amplified by PCR using cDNA template. Then, the amplified sequences and the sequences of circ_0114428 and ROCK2 3ʹUTR with nucleotide substitutions in their putative binding sites were introduced into the pmirGLO vector to build wild-type (WT) and mutant (MUT) plasmids, including WT-circ_0114428, MUT-circ_0114428, WT-ROCK2 3’UTR and MUT-ROCK2 3ʹUTR. HPAEpiCs were co-transfected with these plasmids and miR-574-5p mimics (or miR-NC). At 48 h post-transfection, luciferase activities were analyzed using a luminometer with a Dual-Lucy Assay Kit (Solarbio).

### RNA immunoprecipitation (RIP)

According to the instruction of Magna RNA immunoprecipitation kit (Millipore, Billerica, MA, USA), HPAEpiCs were lysed and miRNA ribonucleoprotein complex coimmunoprecipitated with AgO2 or IgG was enriched. Co-precipitated RNAs induced by anti-AgO2 (1:50; Abcam) or anti-IgG (1:100; Abcam) were analyzed by qRT-PCR.

### RNA pull-down assay

MiR-574-5p and miR-NC were biotinylated to generate bio-miR-574-5p and bio-miR-NC by GenePharma Company (Shanghai, China), and then these biotinylated oligonucleotides were transfected into HPAEpiCs for 48 h. Subsequently, the cells were collected and lysed, and the lysates were incubated with streptavidin-coated magnetic beads. The biotin-coupled RNA complex was pulled down and then was analyzed by qRT-PCR.

### Statistical analysis

The data from three independent duplicate tests were analyzed on GraphPad Prism or image J software. The results were shown as means ± standard deviations. The comparisons for significant differences were performed with Student’s *t* tests, Wilcoxon rank-sum test, Spearman’s correlation test or analysis of variance. *P* < 0.05 was considered statistically significant.

## Results

### Circ_0114428 expression is upregulated in the blood samples of patients with sepsis and LPS-induced HPAEpiCs

**Fig. 1:**
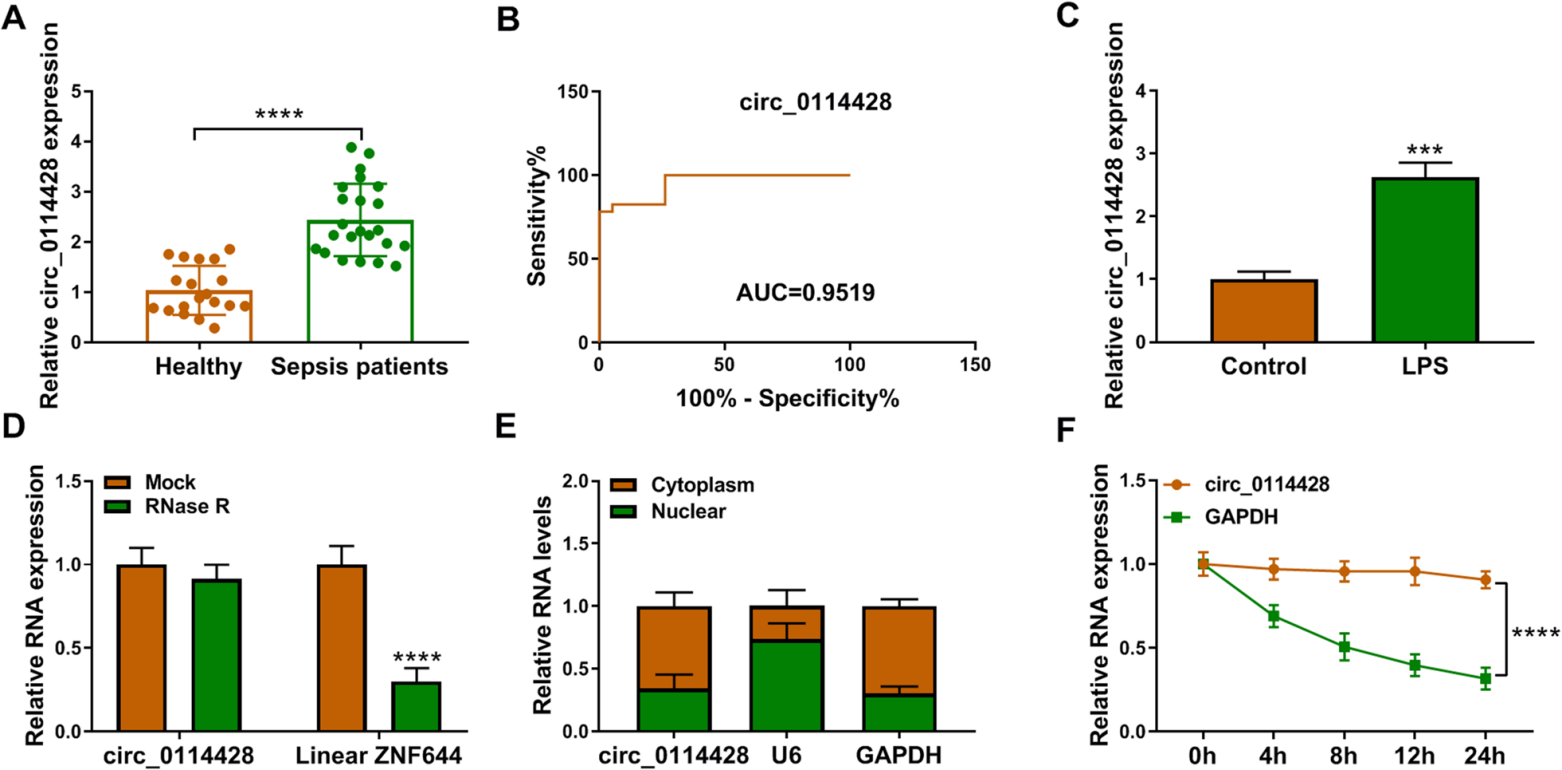
The expression of circ_0114428 in sepsis patients and LPS-treated HPAEpiCs. **A** Circ_0114428 expression was detected by qRT-PCR in blood samples from healthy volunteers (*N* = 19) and sepsis patients (*N* = 23). **B** Receiver Operating Characteristic (ROC) curve analysis of the diagnostic value of circ_0114428 in ALI. **C** Circ_0114428 expression was detected by qRT-PCR in LPS-stimulated HPAEpiCs and untreated HPAEpiCs. **D** RNase R treatment assay was used to identify the stability of circ_0114428. **E** Subcellular fractionation assay was performed to demonstrate that circ_0114428 was mainly localized in the cytoplasm. **F** The circular structure of circ_0114428 was assessed by Actinomycin D assay. ***P < 0.001 and ****P < 0.0001

Circ_0114428 is located on the chr1:91403041–91447927 and formed by the cyclization of the exons 2–4 of the ZNF644 gene with a spliced length of 3705 nt (Additional file 1: Figure S1). Twenty-three blood samples and nineteen healthy blood samples were collected from sepsis patients and volunteers, respectively and were then subjected to qRT-PCR analysis to determine circ_0114428 expression profile. As shown in Fig. 1A, circ_0114428 was significantly increased in the samples from sepsis patients in comparison with its expression in volunteers. As shown in Additional file 2: Figure S2, circ_0114428 expression was significantly increased in the serum of ALI patients with sepsis when compared with healthy volunteers and ALI patients without sepsis. We also found that circ_0114428 had high diagnostic value in ALI with an AUC of 0.9519 (Fig. 1B). The study found that circ_0114428 was overexpressed in LPS-stimulated HPAEpiCs, when compared with controls (Fig. 1C). Subsequently, it was shown that circ_0114428 was resistant to RNase R treatment, although linear ZNF644 expression was greatly downregulated (Fig. 1D), which suggested the high stability of circ_0114428. Consistently, the subcellular fractionation assay revealed that circ_0114428 was mainly localized in the cytoplasm (Fig. 1E). As shown in Fig. 1F, the transcript half-life of circ_0114428 exceeded 24 h, but that of linear GAPDH was about 8 h. These findings demonstrated that circ_0114428 might be associated with sepsis-induced ALI.

### Circ_0114428 knockdown assuages LPS-induced HPAEpiC injury

**Fig. 2:**
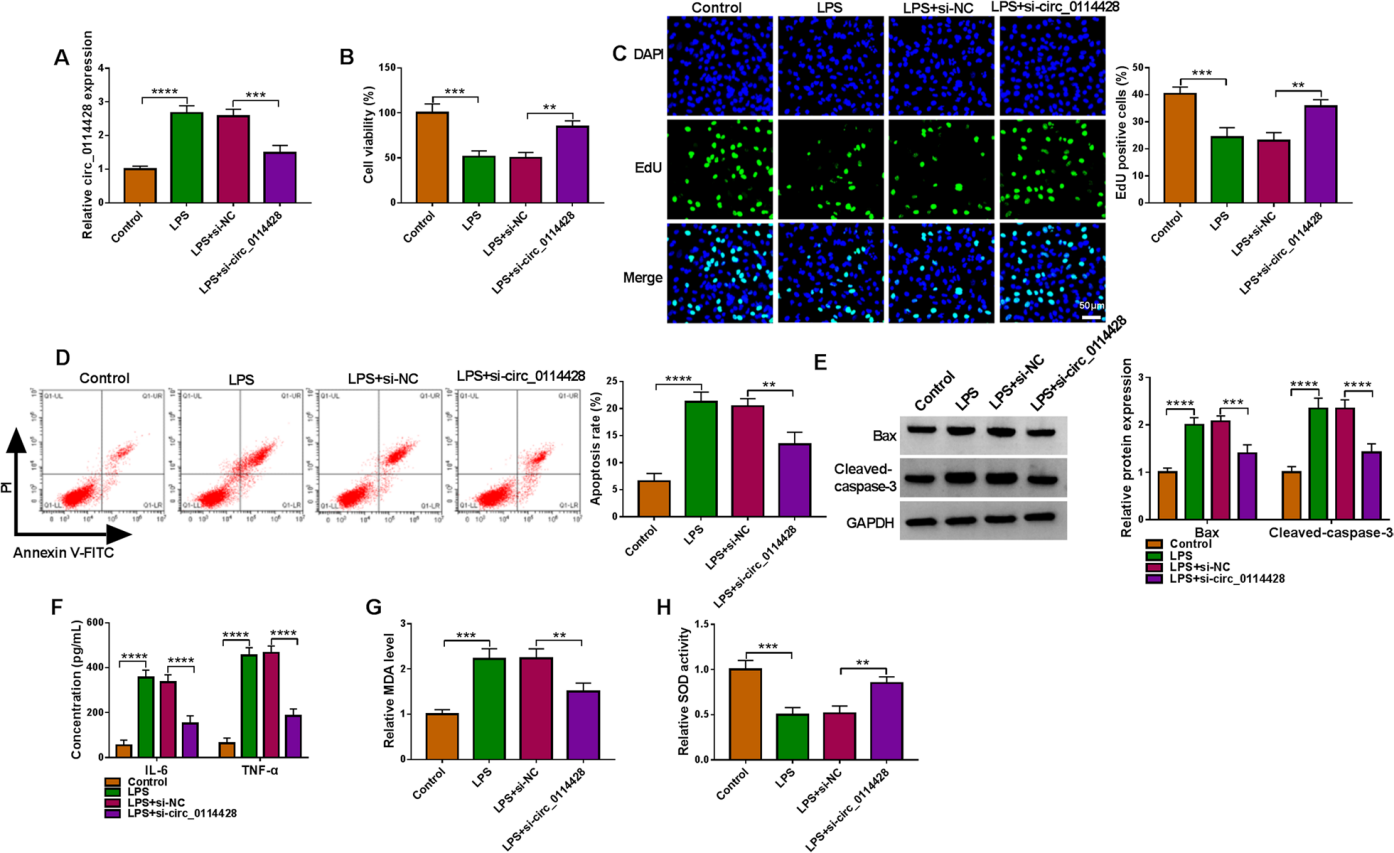
The regulation of circ_0114428 towards LPS-induced HPAEpiC disorders. HPAEpiCs were treated with LPS, LPS+si-NC, or LPS+si-circ_0114428, with untreated HPAEpiCs as a control, and circ_0114428 expression was determined by qRT-PCR (**A**), cell viability by CCK-8 assay (**B**), cell proliferation by EdU assay (**C**), cell apoptosis by flow cytometry analysis (**D**), the protein expression of Bax and cleaved-caspase-3 by Western blotting analysis (**E**), the secretion of IL-6 and TNF-α by ELISA (**F**), MDA level by lipid peroxidation MDA assay (**G**), and SOD activity by SOD activity detection assay (**H**). **P < 0.01, ***P < 0.001 and ****P < 0.0001

We then transfected the siRNA for circ_0114428 into LPS-treated HPAEpiCs to determine its possible role in sepsis-induced ALI. Results first showed that LPS treatment upregulated circ_0114428 expression, whereas the effect was relieved after circ_0114428 knockdown (Fig. 2A). Subsequently, LPS treatment inhibited HPAEpiC viability and proliferation, which were remitted when circ_0114428 was decreased (Fig. 2B and C). As presented in Fig. 2D and E, LPS stimulation induced cell apoptosis, accompanied by the increases of Bax and cleaved-caspase-3 expression; however, these effects were remitted after the combined treatment of LPS and si-circ_0114428. Besides, circ_0114428 silencing counteracted LPS-triggered inflammatory response and oxidative stress. For instance, LPS treatment stimulated the production of IL-6, TNF-α and MDA, and inhibited SOD activity, but these effects were relieved after the knockdown of circ_0114428 (Fig. 2F–H). Collectively, all findings confirmed that circ_0114428 participated in the regulation of LPS-induced lung cell injury.

### Circ_0114428 acts as a miR-574-5p sponge

**Fig. 3:**
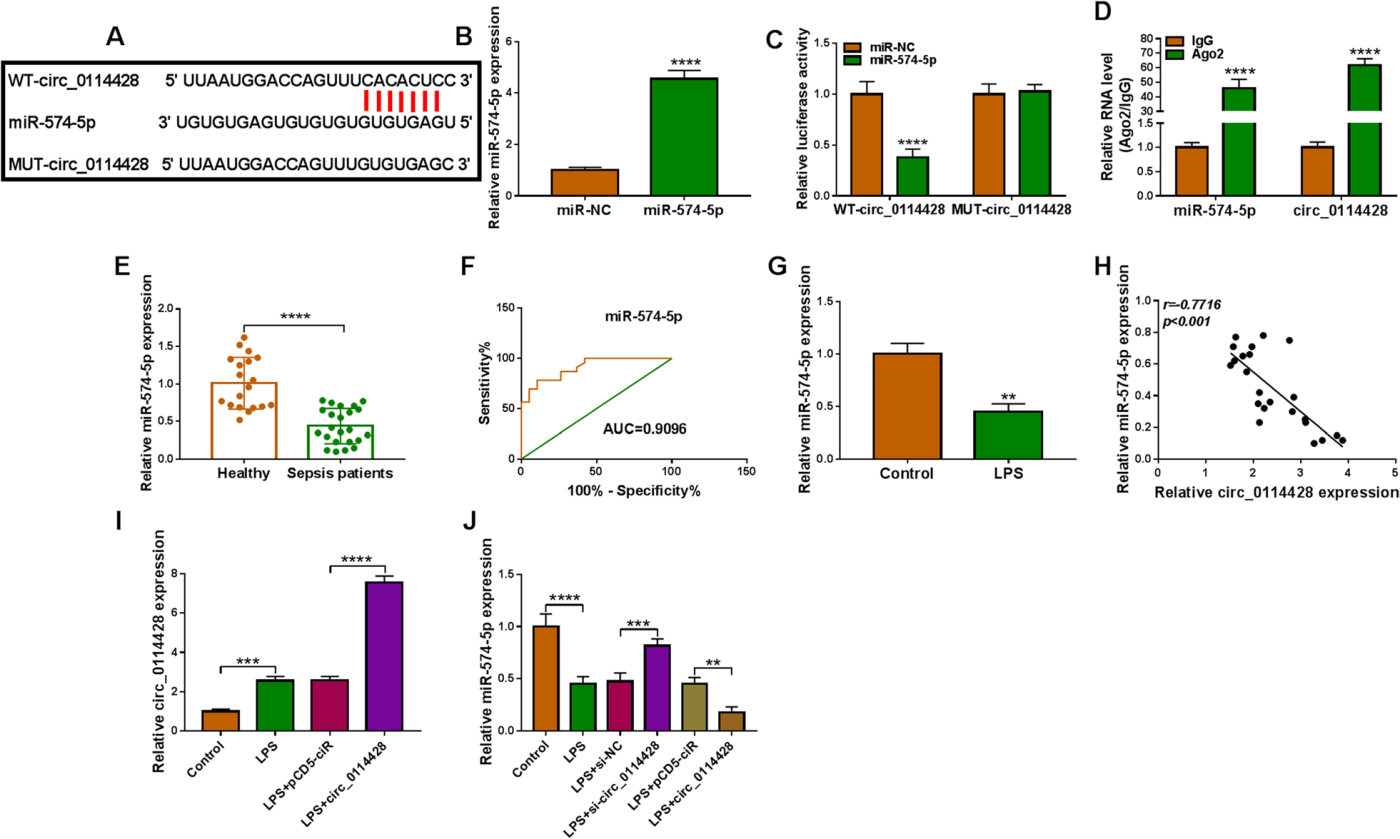
Circ_0114428 combines with miR-574-5p. **A** Schematic illustration showing miR-574-5p-binding sites on circ_0114428 and the mutated sites of circ_0114428. **B** The efficiency of miR-574-5p overexpression was detected by qRT-PCR. **C**, **D** Dual-luciferase reporter and RIP assays were employed to demonstrate the interplay of circ_0114428 and miR-574-5p. **E** MiR-574-5p expression was detected by qRT-PCR in blood samples from healthy volunteers (*N* = 19) and blood samples from sepsis patients (*N* = 23). **F** ROC curve analysis of the diagnostic value of miR-574-5p in ALI. **G** MiR-574-5p expression was detected by qRT-PCR in LPS-stimulated HPAEpiCs and untreated HPAEpiCs. **H** Spearman correlation analysis was carried out to determine the linear correlation between miR-574-5p and circ_0114428. **I** The efficiency of circ_0114428 overexpression was detected by qRT-PCR in LPS-treated HPAEpiCs. **J** The impact of circ_0114428 knockdown or overexpression on LPS-mediated miR-574-5p expression was determined by qRT-PCR. **P < 0.01, ***P < 0.001 and ****P < 0.0001

To reveal the mechanism underlying circ_0114428 regulating LPS-caused lung cell injury, its miRNA target was predicted by circinteractome online database. Given many miRNAs with potential binding sites of circ_0114428. The study analyzed miRNAs that were lowly expressed in sepsis-induced ALI patients and inhibited the progression of the disease. As shown in Additional file 3: Figure S3A, circ_0114428 depletion significantly increased miR-142-5p and miR-574-5p expression, especially miR-574-5p expression. Thus, miR-574-5p was chosen for the following study. As displayed in Fig. 3A, miR-574-5p, a candidate, contained the binding sites of circ_0114428. To determine whether circ_0114428 combined with miR-574-5p, miR-574-5p was co-transfected into HPAEpiCs with reporter plasmids (WT-circ_0114428 and MUT-circ_0114428). The success of miR-574-5p overexpression was shown in Fig. 3B. Then, we found that enforced expression of miR-574-5p significantly inhibited the luciferase activity of WT-circ_0114428 rather than that of mutant (Fig. 3C). Meanwhile, the RIP assay showed that the antibody specific to Ago2 greatly enriched both miR-574-5p and circ_0114428 when compared with controls (Fig. 3D). The RNA pull-down assay showed that circ_0114428 was significantly enriched in the bio-miR-574-5p group in comparison with the bio-miR-NC group (Additional file 4: Figure S4). These findings confirmed the role of circ_0114428 as a miR-574-5p sponge. Consistently, sepsis patients and LPS-stimulated HPAEpiCs showed a low expression of miR-574-5p when compared with control groups (Fig. 3E and G). We also found that miR-574-5p had high diagnostic value in ALI with an AUC of 0.9096 (Fig. 3F). The association between circ_0114428 and miR-574-5p expression in the blood samples from sepsis patients was analyzed by Spearman’s correlation analysis. As expected, circ_0114428 was negatively correlated with miR-574-5p in expression (Fig. 3H). Furthermore, the effect of circ_0114428 on LPS-reduced miR-574-5p expression was determined by qRT-PCR. Before that, we demonstrated the success of circ_0114428 overexpression in LPS-treated HPAEpiCs (Fig. 3I). Subsequent data showed that LPS-induced inhibition of miR-574-5p expression was restored by circ_0114428 downregulation, but enhanced by circ_0114428 upregulation (Fig. 3J).

### Circ_0114428 regulates LPS-induced lung cell injury through miR-574-5p

**Fig. 4:**
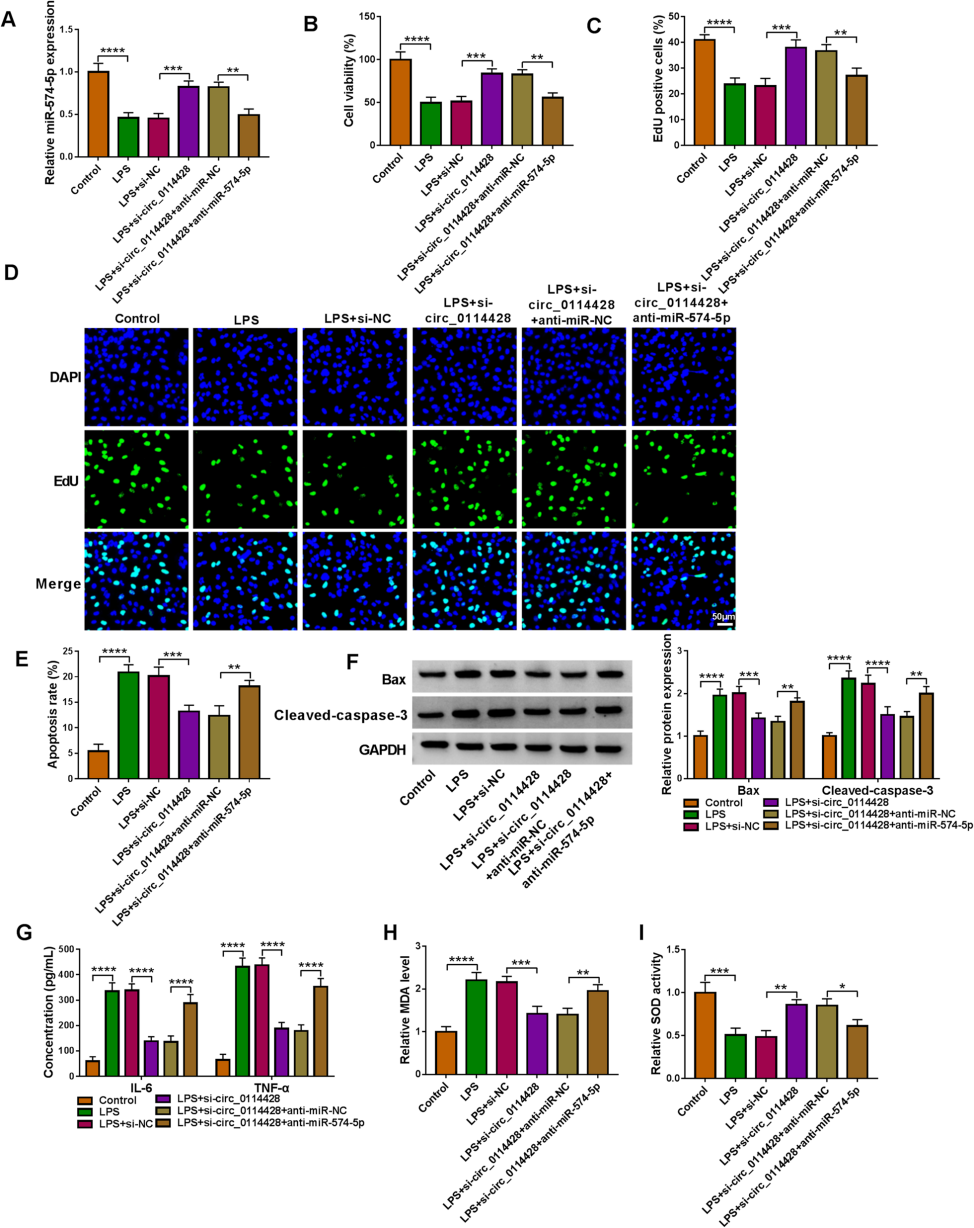
The effects between circ_0114428 knockdown and miR-574-5p silencing on LPS-induced HPAEpiC injury. HPAEpiCs were treated with LPS, LPS+si-NC, LPS+si-circ_0114428, LPS+si-circ_0114428+anti-miR-NC or LPS+si-circ_0114428+anti-miR-574-5p, with mock HPAEpiCs as a control, and miR-574-5p expression was determined by qRT-PCR (**A**), cell viability by CCK-8 assay (**B**), cell proliferation by EdU assay (**C** and **D**), cell apoptosis by flow cytometry analysis (**E**), the protein expression of Bax and cleaved-caspase-3 by Western blotting analysis (**F**), the secretion of IL-6 and TNF-α by ELISA (**G**), MDA level by lipid peroxidation MDA assay (**H**), and SOD activity by SOD activity detection assay (**I**). *P < 0.05, **P < 0.01, ***P < 0.001 and ****P < 0.0001

Si-circ_0114428, si-circ_0114428 + anti-miR-574-5p and matched controls were transfected into LPS-stimulated HPAEpiCs to determine whether miR-574-5p participated in the regulation of circ_0114428 towards LPS-caused cell injury. The results first showed the inhibitory effect of circ_0114428 knockdown on LPS-reduced miR-574-5p expression was relieved after transfection with anti-miR-574-5p (Fig. 4A). Subsequently, circ_0114428 knockdown-increased cell viability and cell proliferation were rescued after miR-574-5p silencing under LPS treatment (Fig. 4B–D). Consistently, circ_0114428 knockdown-reduced cell apoptosis and the protein expression of Bax and cleaved-caspase-3 were remitted when miR-574-5p was downregulated (Fig. 4E and F). Similarly, the decreased production of IL-6, TNF-α and MDA and increased SOD activity by circ_0114428 knockdown were counteracted after transfection with miR-574-5p inhibitors (Fig.4G–I). Collectively, these findings suggested that circ_0114428 knockdown assuaged LPS-induced lung cell disorders by binding to miR-574-5p.

### MiR-574-5p targets ROCK2 in HPAEpiCs

**Fig. 5:**
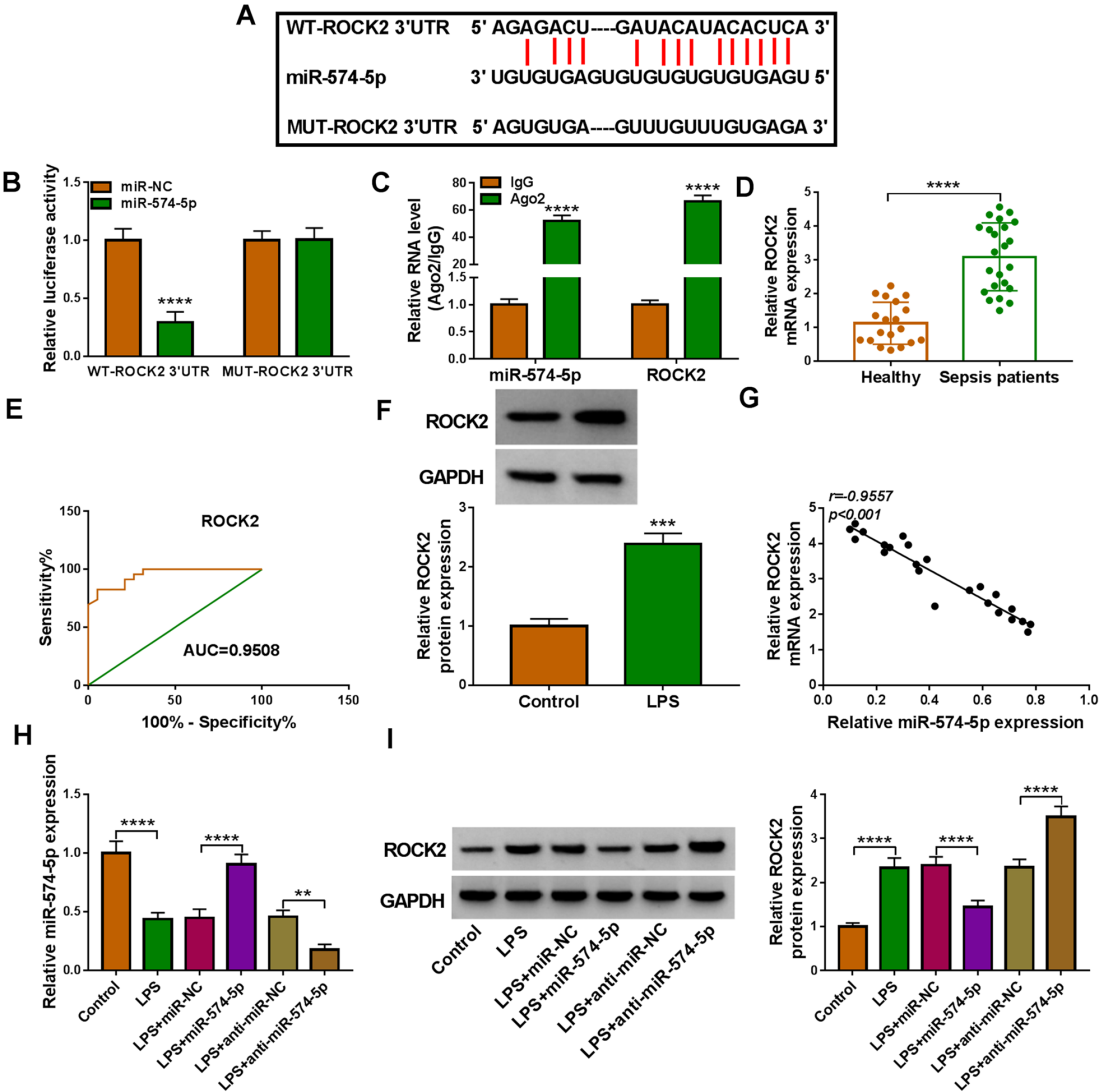
MiR-574-5p combines with ROCK2. **A** Schematic illustration showing miR-574-5p-binding sites in ROCK2 3’UTR. **B**, **C** The interplay between miR-574-5p and ROCK2 was confirmed by dual-luciferase reporter assay and RIP assay. **D** ROCK2 expression was detected by qRT-PCR in blood samples from healthy volunteers and blood samples from sepsis patients. **E** ROC curve analysis of the diagnostic value of ROCK2 in ALI. **F** MiR-574-5p expression was detected by Western blotting analysis in LPS-stimulated HPAEpiCs and untreated HPAEpiCs. **G** Spearman correlation analysis was carried out to determine the linear correlation between miR-574-5p and ROCK2 expression in the blood samples from sepsis patients. **H** The efficiency of miR-574-5p overexpression or knockdown was determined by qRT-PCR in LPS-treated HPAEpiCs. **I** The effects of miR-574-5p overexpression and knockdown on LPS-increased ROCK2 production were determined by Western blotting. **P < 0.01, ***P < 0.001 and ****P < 0.0001

Starbase online database was performed to predict the targeting gene of miR-574-5p. The study analyzed the target genes that were highly expressed in sepsis-induced ALI patients and promoted the development of the disease. As shown in Additional file 3: Figure S3B, miR-574-5p overexpression significantly decreased TLR4, MYD88 and ROCK2 expression, especially ROCK2 expression. Thus, ROCK2 was chosen for the following study. As displayed in Fig. 5A, the complementary sites of miR-574-5p were found in ROCK2 3ʹUTR, showing the potential of ROCK2 as a miR-574-5p target. To confirm the hypothesis, dual-luciferase reporter and RIP assays were conducted. As expected, miR-574-5p overexpression significantly decreased the luciferase activity of wild-type reporter plasmid of ROCK2 3ʹUTR, whereas the luciferase activity of mutant had no response to miR-574-5p overexpression (Fig. 5B). Moreover, the antibody against Ago2 dramatically enriched both miR-574-5p and ROCK2, when compared with controls (Fig. 5C). Based on the above results, ROCK2 was chosen as a targeting gene of miR-574-5p. Comparatively, ROCK2 was significantly upregulated in the blood samples of sepsis patients and LPS-treated HPAEpiCs (Fig. 5D and F). ROCK2 had high diagnostic value in ALI with an AUC of 0.9508 (Fig. 3E). As revealed by Spearman correlation analysis, miR-574-5p was negatively correlated with ROCK2 in expression in the blood samples of sepsis patients (Fig. 5G). After determining the success of miR-574-5p overexpression or knockdown in LPS-treated HPAEpiCs (Fig. 5H), we explored the effects of both miR-574-5p upregulation and downregulation on LPS-increased ROCK2 expression. As shown in Fig. 5I, the promoting effect of LPS on ROCK2 expression was relieved after miR-574-5p overexpression, but enhanced after transfection with miR-574-5p inhibitors. Thus, all findings demonstrated that miR-574-5p bound to ROCK2.

### ROCK2 knockdown ameliorates LPS-induced lung cell dysfunction

**Fig. 6:**
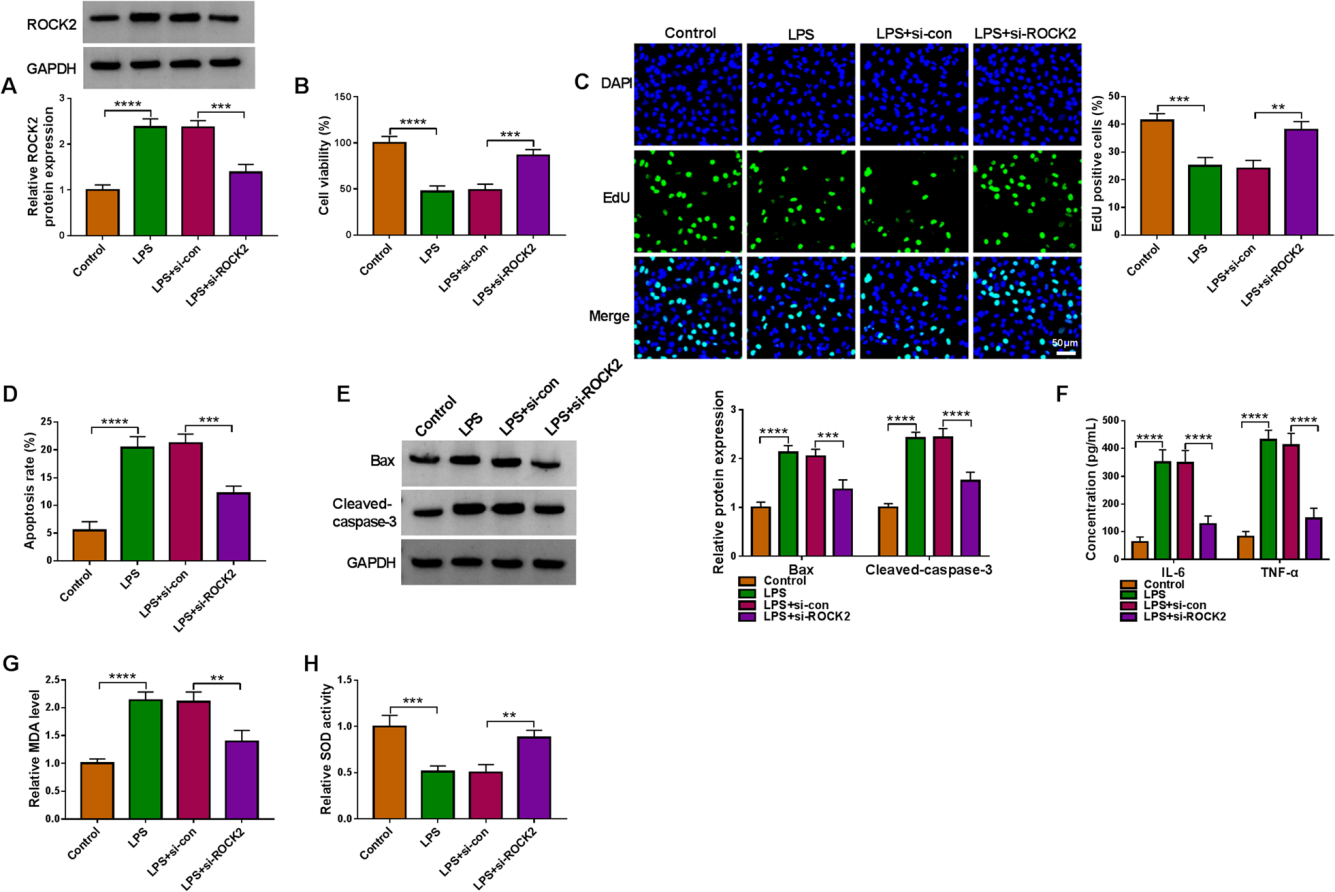
The effect of ROCK2 knockdown in LPS-induced HPAEpiC injury. HPAEpiCs were treated with LPS, LPS+si-con, or LPS+si-ROCK2, with untreated HPAEpiCs as a control, and ROCK2 expression was determined by Western blotting (**A**), cell viability by CCK-8 assay (**B**), cell proliferation by EdU assay (**C**), cell apoptosis by flow cytometry analysis (**D**), the protein expression of Bax and cleaved-caspase-3 by Western blotting analysis (**E**), the secretion of IL-6 and TNF-α by ELISA (**F**), MDA level by lipid peroxidation MDA assay (**G**), and SOD activity by SOD activity detection assay (**H**). **P < 0.01, ***P < 0.001 and ****P < 0.0001

Given the targeting relationship of miR-574-5p and ROCK2, it was inferred that ROCK2 was associated with LPS-induced cell disorders. To prove the hypothesis, the siRNA specific to ROCK2 was transfected into LPS-stimulated HPAEpiCs. Figure 6A showed the inhibitory effect of ROCK2 knockdown on LPS-increased ROCK2 production. Then, we found that LPS-induced inhibition of cell viability and cell proliferation was attenuated after ROCK2 knockdown (Fig. 6B and C). It was found using flow cytometry analysis and Western blotting that the increased cell apoptosis and the expression of Bax and cleaved-caspase-3 were restored when ROCK2 was decreased (Fig. 6D and E). Consistently, LPS treatment promoted the production of IL-6, TNF-α and MDA, and inhibited SOD activity; however, these effects were remitted by ROCK2 downregulation (Fig. 6F–H). All in all, these results showed that ROCK2 was a mediator of LPS-induced lung cell disorders, which exhibited a similar effect as did circ_0114428 in LPS-induced lung cells.

### MiR-574-5p modulates LPS-induced HPAEpiC injury by combining with ROCK2

**Fig. 7:**
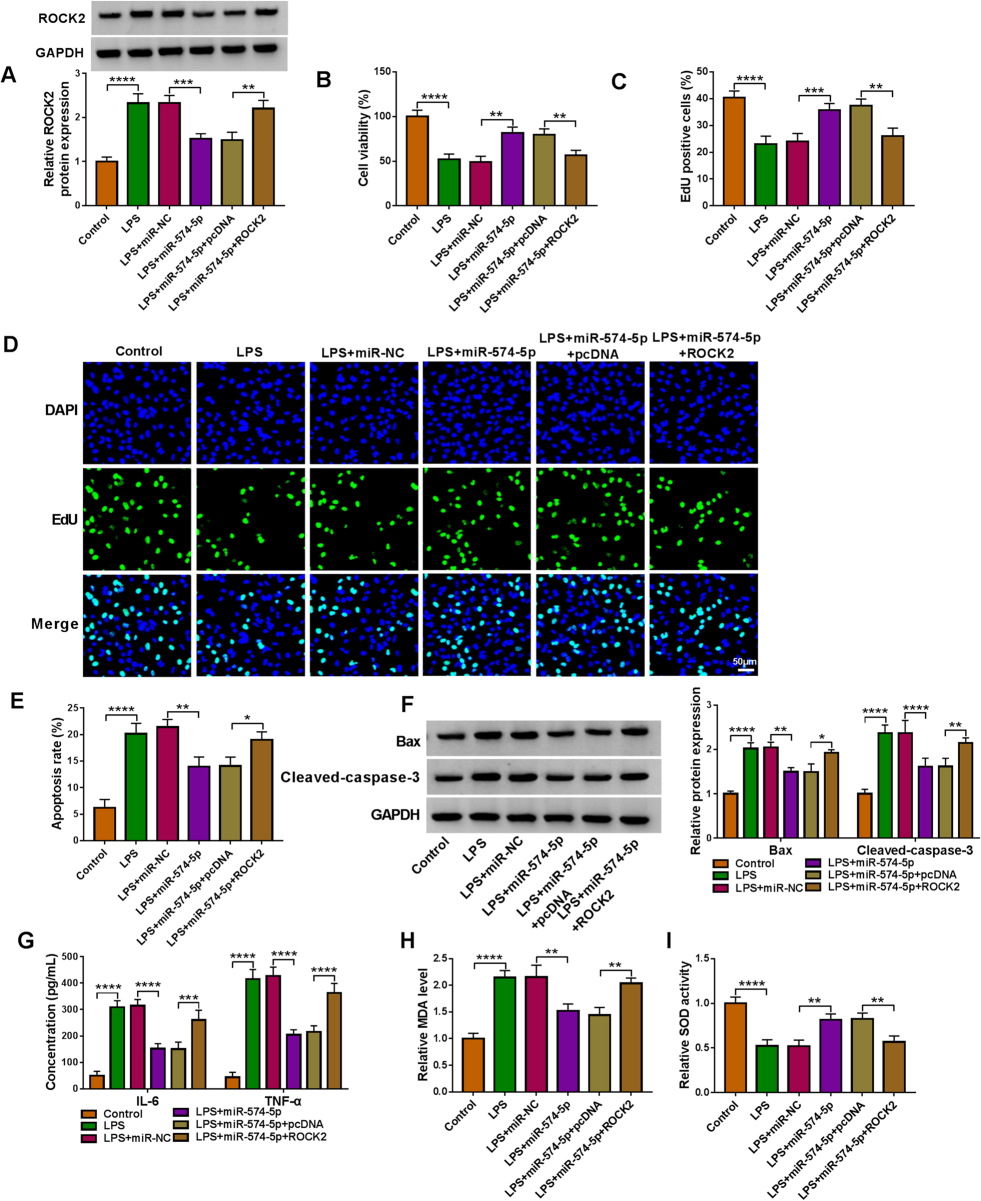
The effects between miR-574-5p and ROCK2 overexpression on LPS-caused cell injury. HPAEpiCs were treated with LPS, LPS+miR-NC, LPS+miR-574-5p, LPS+miR-574-5p+pcDNA or LPS+miR-574-5p+ROCK2, with untreated HPAEpiCs as a control, and ROCK2 expression was determined by Western blotting (**A**), cell viability by CCK-8 assay (**B**), cell proliferation by EdU assay (**C** and **D**), cell apoptosis by flow cytometry analysis (**E**), the protein expression of Bax and cleaved-caspase-3 by Western blotting analysis (**F**), the secretion of IL-6 and TNF-α by ELISA (**G**), MDA level by lipid peroxidation MDA assay (**H**), and SOD activity by SOD activity detection assay (**I**). *P < 0.05, **P < 0.01, ***P < 0.001 and ****P < 0.0001

Based on the above results, we continued to explore whether ROCK2 was involved in miR-574-5p-mediated action in LPS-induced HPAEpiCs. To this end, we overexpressed both miR-574-5p and ROCK2 in the cells. The data from Fig. 7A exhibited that miR-574-5p decreased ROCK2 expression under LPS treatment, which was attenuated after ROCK2 overexpression. Subsequently, miR-574-5p-induced promotion of cell viability and cell proliferation was restored by enforced expression of ROCK2 in LPS-treated HPAEpiCs (Fig. 7B–D). Consistently, the decreased cell apoptosis and the protein expression of Bax and cleaved-caspase-3 were remitted when ROCK2 was upregulated (Fig. 7E and F). Similarly, miR-574-5p-induced inhibition of IL-6, TNF-α and MDA levels and promotion of SOD activity were rescued after ROCK2 upregulation (Fig. 7G–I). Thus, these findings demonstrated the involvement of miR-574-5p/ROCK2 pathway in LPS-induced lung cell injury.

### Circ_0114428 knockdown inhibits ROCK2 production by associating with miR-574-5p

**Fig. 8:**
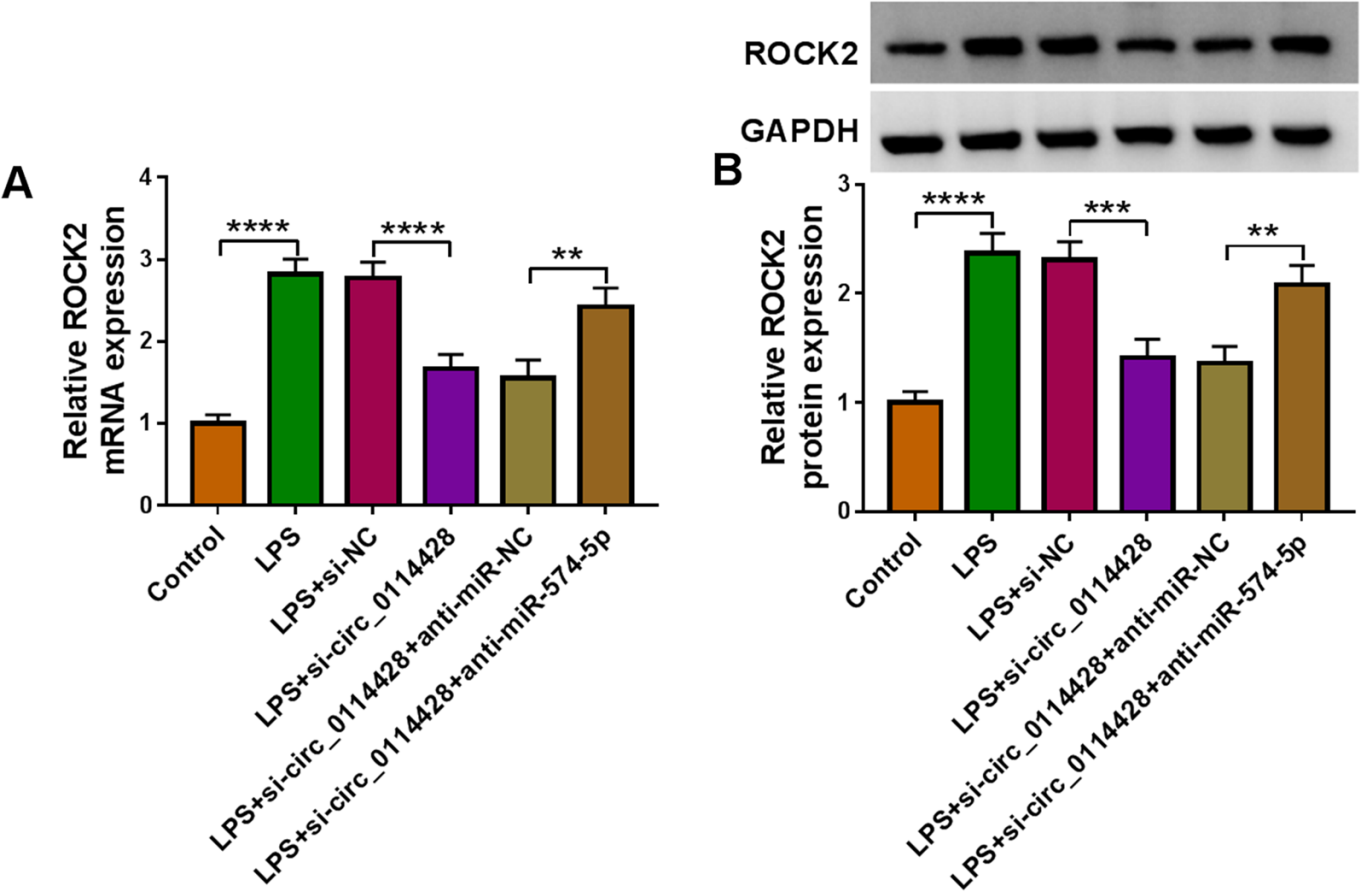
The effects between circ_0114428 silencing and miR-574-5p knockdown on ROCK2 expression. HPAEpiCs were treated with LPS, LPS+si-NC, LPS+si-circ_0114428, LPS+si-circ_0114428+anti-miR-NC or LPS+si-circ_0114428+anti-miR-574-5p, with mock HPAEpiCs as a control. ROCK2 mRNA expression was analyzed by qRT-PCR (**A**). ROCK2 protein expression was detected by Western blotting (**B**). **P < 0.01, ***P < 0.001 and ****P < 0.0001

Based on the above findings, we hypothesized that circ_0114428 regulated ROCK2 through miR-574-5p. To prove this speculation, we silenced both circ_0114428 and miR-574-5p in LPS-stimulated HPAEpiCs and then detected ROCK2 expression by qRT-PCR and Western blotting. As shown in Fig. 8A and B, circ_0114428 knockdown reduced the mRNA and protein expression of ROCK2, which was attenuated when miR-574-5p was decreased, proving our conjecture.

## Discussion

Defined as a polar lipid head and chain repeating disaccharide, LPS is the major component of Gram-negative bacteria. LPS is an important medium for sepsis and can activate LPS-binding protein complex of CD14/TLR4 receptor on some types of cells, such as monocytes, and macrophages, thereby inducing the production of inflammatory mediators [20]. The recent data explained that LPS treatment induced lots of polymorphonuclear leucocyte migration into mouse lungs, causing typical ALI symptoms, which included the increase of microvascular permeability, the production of cytokine as well as the damage of lung structure [21]. As a result, LPS is commonly used to mimic sepsis-induced lung injury model [22, 23]. Based on this model, this work explored the role of circ_0114428 in sepsis-induced ALI, and found that circ_0114428 knockdown protected against LPS-caused lung cell damage. Subsequent results demonstrated that the effect of circ_0114428 on LPS-induced cell injury involved miR-574-5p and ROCK2.

CircRNA is a noncoding RNA that plays important roles in disease development. Recent studies confirmed the involvement of circRNA in sepsis-induced ALI. For example, Bao and his colleagues indicated that there were 137 differently expressed circRNAs in the macrophages from lung homogenates after cecal ligation and puncture (CLP) treatment, and subsequent experiments predicted the association of these upregulated circRNAs with mitochondrion distribution and Notch binding [24]. Zou et al. identified the upregulation of circ_0001679 and circ_0001212 in LPS-induced mice, and these two circRNAs participated in the regulation of pulmonary P2X7 receptor towards sepsis-induced ALI [25]. In another work, the overexpression of circRNA complement component 3 precursor pseudogene (circC3P1) in CLP-induced ALI mice assuaged lung damage, inflammation and apoptosis [26]. In the present work, we disclosed the role of another circRNA, circ_0114428, in sepsis-induced ALI for the first time. We found that circ_0114428 was upregulated in LPS-stimulated lung cells. Moreover, circ_0114428 expression was significantly increased in the serum of ALI patients with sepsis when compared with healthy volunteers and ALI patients without sepsis, which suggested that circ_0114428 upregulation were primarily attributed to the development of ALI as a consequence of sepsis. Circ_0114428 had a high diagnostic value in ALI. The knockdown of circ_0114428 in LPS-treated lung cells attenuated LPS-induced inhibition of cell proliferation and promotion of cell apoptosis, inflammation response and oxidative stress. Our data suggested the protective role of circ_0114428 knockdown in LPS-induced lung injury.

Given that circRNA could function by sponging miRNA, the miRNA able to combine with circ_0114428 was analyzed. The previous results have indicated the association of miR-574-5p (also termed miR-574) with the progression of some diseases, such as cancer [27], cardiac fibroblasts [28] and coronary artery disease [14]. The recent investigation also testified the decreased expression of miR-574-5p in sepsis patients with AKI, and the miRNA was associated with kidney injury biomarker [29]. Our work further unveiled the downregulation of miR-574-5p in the blood samples of sepsis patients and LPS-stimulated lung cells. Besides, miR-574-5p had antagonizing effects against circ_0114428. In the work of Sun et al., it was shown that miR-574-5p regulated LPS- or CLP-induced sepsis lung injury through interaction with Complement 3 (C3) [30]. Herein, ROCK2 was identified as a miR-574-5p target.

ROCK2 is a serine–threonine kinase that is an important regulator in Rho/ROCK signaling pathway [31]. Some studies have indicated the upregulation of ROCK2 in the development of cancers and noncancer diseases [32]. In particular, ROCK2 was associated with lung injury. Chen et al. indicated that ROCK2 enhanced LPS-caused inflammation and apoptosis in LPS-treated MPVECs [33]. Fasudil-induced inhibition of RHO assuaged ischemic lung injury by reducing both ROCK2 and ROCK1 in a rat model [34]. Hydrogen-induced inhibition of ALI in sepsis mice involved ROCK2 [35]. In this work, sepsis patients and LPS-treated lung cells showed a high expression of ROCK2. ROCK2 downregulation displayed protective effects against LPS-induced lung cell injury. In addition, miR-574-5p regulated LPS-caused cell disorders through binding to ROCK2. Importantly, circ_0114428 mediated ROCK2 production by combing with miR-574-5p.

However, the mechanism responsible for ROCK2-mediated effects in LPS-induced lung cells remains absent in this work, and further investigation in this regard should be performed in the future. Besides, the inhibitory effects of circ_0114428 silencing on LPS-triggered lung cell dysfunctions are only analyzed in vitro, and mouse model assay should be performed to testify the results from in vitro assay. Further, in addition to the circ_0114428/miR-574-5p/ROCK2 pathway, other regulatory pathways, such as circ_0001679/miR-338-3p/DUSP16 [36] and circPALM2/miR-330-5p/ROCK2 [37], also were involved in the occurrence of ALI, which suggested that the circ_0114428/miR-574-5p/ROCK2 axis was but one of the pathways related to ALI occurrence. The above issues should be considered when evaluating the present study.

Collectively, LPS-caused lung cell injury involved the upregulation of circ_0114428. The increased expression of circ_0114428 negatively regulated miR-574-5p expression to induce ROCK2 expression, thereby leading to inhibition of cell proliferation, and promotion of cell apoptosis, inflammation and oxidative stress (Additional file 5: Figure S5). Consequentially, inhibiting circ_0114428 expression might be a possible strategy for the therapy of sepsis-induced ALI.

### Supplementary Information

**Additional file 1: Figure S1.** The schematic diagram shows the formation of circ_0114428.

**Additional file 2: Figure S2.** qRT-PCR is used to analyze circ_0114428 expression in the serum of ALI patients with sepsis, ALI patients without sepsis and healthy volunteers. ****P < 0.0001.

**Additional file 3: Figure S3.** qRT-PCR is used to assess the expression levels of circ_0114428-associated miRNAs and miR-574-5p-associated mRNAs. *P < 0.05 and ****P < 0.0001.

**Additional file 4: Figure S4.** RNA pull-down assay is performed to analyze the association between circ_0114428 and miR-574-5p. ***P < 0.001.

**Additional file 5: Figure S5.** A model diagram that illustrates the mechanism of circ_0114428 in regulating lipopolysaccharide-induced human pulmonary alveolar epithelial cell injury.

## Data Availability

The analyzed data sets generated during the present study are available from the corresponding author on reasonable request.
