# Supplementary figures and images for: Circ_0114428 knockdown inhibits ROCK2 expression to assuage lipopolysaccharide-induced human pulmonary alveolar epithelial cell injury through miR-574-5p
Source: J Physiol Sci. 2024 Jan 31;74:5. doi: 10.1186/s12576-023-00891-3 (PMC10829305; doi:10.1186/s12576-023-00891-3)

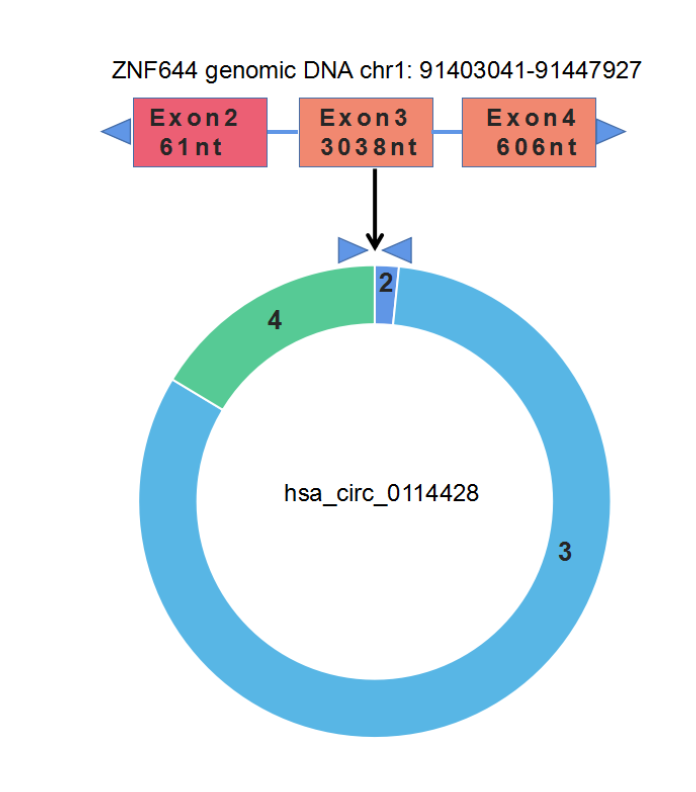

Supplement: Supplementary file 1 — Additional file 1: Figure S1. The schematic diagram shows the formation of circ_0114428. [file 12576_2023_891_MOESM1_ESM.tif]

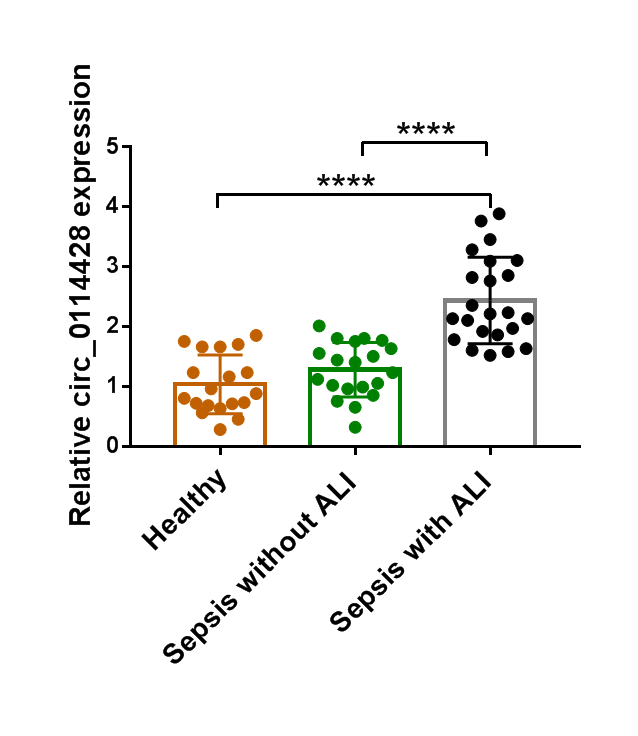

Supplement: Supplementary file 2 — Additional file 2: Figure S2. qRT-PCR is used to analyze circ_0114428 expression in the serum of ALI patients with sepsis, ALI patients without sepsis and healthy volunteers. ****P < 0.0001. [file 12576_2023_891_MOESM2_ESM.tif]

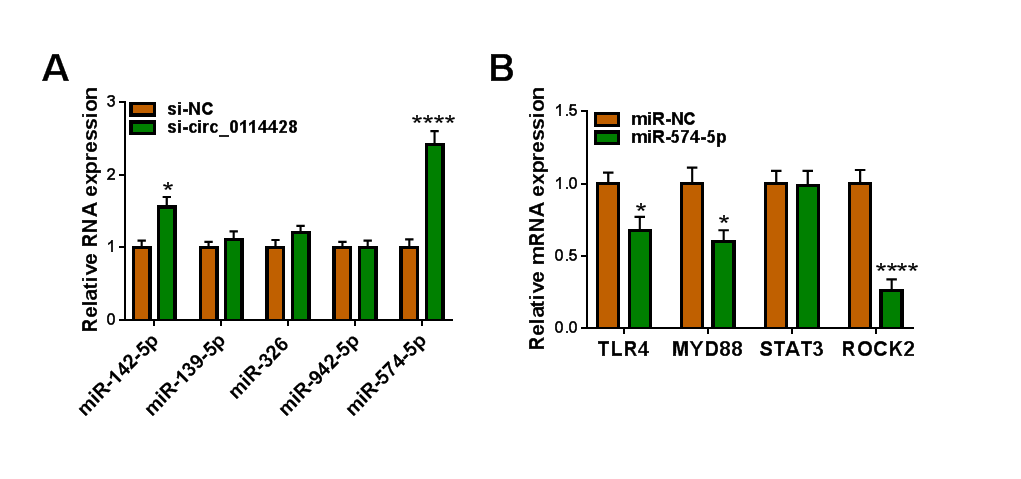

Supplement: Supplementary file 3 — Additional file 3: Figure S3. qRT-PCR is used to assess the expression levels of circ_0114428-associated miRNAs and miR-574-5p-associated mRNAs. *P < 0.05 and ****P < 0.0001. [file 12576_2023_891_MOESM3_ESM.tif]

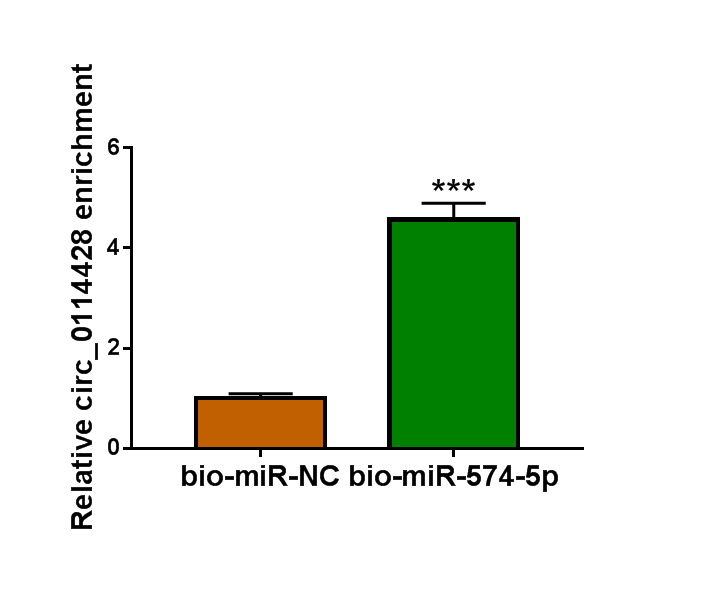

Supplement: Supplementary file 4 — Additional file 4: Figure S4. RNA pull-down assay is performed to analyze the association between circ_0114428 and miR-574-5p. ***P < 0.001. [file 12576_2023_891_MOESM4_ESM.tif]

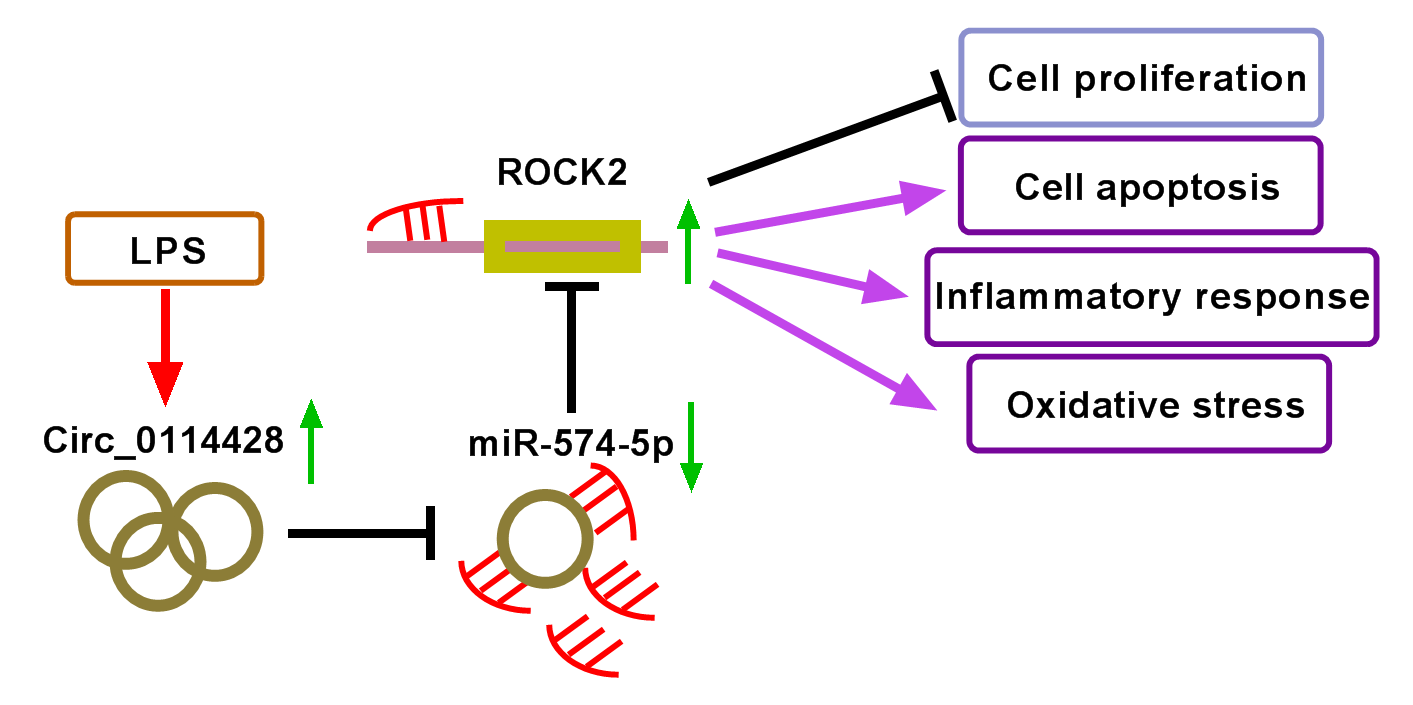

Supplement: Supplementary file 5 — Additional file 5: Figure S5. A model diagram that illustrates the mechanism of circ_0114428 in regulating lipopolysaccharide-induced human pulmonary alveolar epithelial cell injury. [file 12576_2023_891_MOESM5_ESM.tif]
